# Adult-Onset Still’s Disease Mimicking Myositis: A Case Report

**DOI:** 10.7759/cureus.76402

**Published:** 2024-12-26

**Authors:** Mario B Prado, Karen Joy B Adiao

**Affiliations:** 1 Department of Physiology, University of the Philippines Manila, Manila, PHL; 2 Department of Neurosciences, Philippine General Hospital, Manila, PHL

**Keywords:** adult-onset still’s disease, case report, hematuria, myalgia, myositis

## Abstract

The combination of severe myalgia, progressive weakness, and blood in the urine often leads a neurologist to consider myositis. Accordingly, reddish urine may be linked to urine myoglobinuria brought about by muscle destruction. Nevertheless, in a young patient with normal creatine kinase complaining of immobility, adult-onset Still’s disease (AOSD) should be one of the top differentials. We discuss a case of a 25-year-old Filipino male who presented to our clinic with a month's history of progressive, generalized weakness, joint pains, and rashes, accompanied initially by undocumented fever, hematuria, and loss of appetite. C-reactive protein (CRP; >5 mg/L) and erythrocyte sedimentation rate [ESR; 21 mm/hr, normal value: <10 mm/hr] were elevated pre- and post-methylprednisolone pulse therapy (MPPT) (CRP: 211 mg/L; ESR: 125 mm/hr). The extremely high serum ferritin levels (1675.56 ug/L, above the machine detection limit) clinched the diagnosis of AOSD. Early detection of AOSD is cost-effective and highly beneficial, as further workup for myositis involves costly antibody testing and unnecessary invasive muscle biopsies.

## Introduction

Adult-onset Still’s disease (AOSD) is a rare condition characterized by a triad of transient high-grade quotidian or double quotidian fever, evanescent rash, and severe polyarthralgia. It is more common among females and around 75% of cases occur between the ages of 16 and 35 years. While individuals with HLA B17, B18, B35, and D32 are believed to be at risk of having this condition, a concrete association between AOSD and HLA loci has never been established by studies [1]. It is a diagnosis of exclusion, as its activity biological marker, the acute phase reactant serum ferritin, is also elevated in many inflammatory conditions. However, unlike in other rheumatic diseases, serum ferritin in AOSD is extremely elevated, often exceeding more than 1000 ng/ml [1].

Although its specific etiology is unknown, AOSD is believed to be an autoinflammatory disease [1,2]. Commonly, it is preceded by non-specific flu caused by cytomegalovirus, Epstein-Barr virus, echovirus, and rubella among others. Around 56-84% of patients develop myalgia and, in rare cases, it may also damage the kidneys, producing varying signs and symptoms including hematuria secondary to possible AA amyloidosis, collapsing glomerulonephropathy, or thrombotic microangiopathy [3].

The combination of severe myalgia, progressive weakness, and blood in the urine often leads a neurologist to consider myositis, especially if the clinician is not usually exposed to AOSD [4]. Accordingly, reddish urine may be linked to urine myoglobinuria brought about by myonecrosis. Nevertheless, in a young patient with normal creatine kinase (CK MM) despite immobility, myalgia, high-grade fever, and evanescent rash, AOSD should be one of the top differentials.

## Case presentation

**Figure 1:**
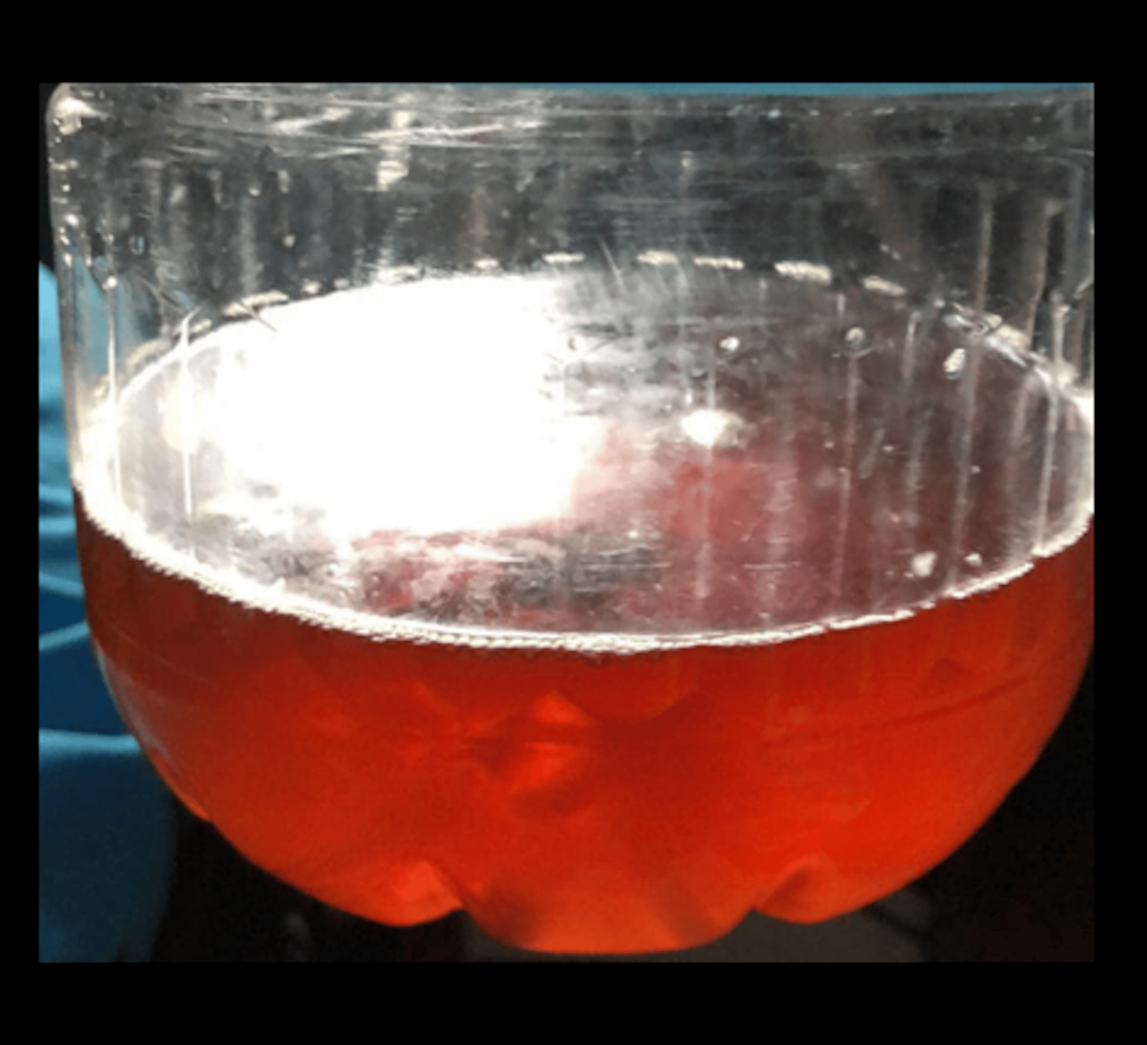
Urinalysis revealing hematuria (abundant red blood cells were found in the sample)

A 25-year-old Filipino male presented to our clinic with a month's history of progressive, generalized weakness, joint pains, and rashes, accompanied initially by undocumented high-grade fever, hematuria, and loss of appetite (Figure 1). Three weeks before the presentation, due to consideration of leptospirosis or myositis, the patient had been admitted for five days and given ceftriaxone and methylprednisolone pulse therapy (MPPT) without tapering, with slight improvement of symptoms.

In the clinic, the patient was bedbound, with unremarkable vital signs and chest and cardiac physical examination findings. He had tender, swollen joints, prominent in all extremities, especially in the lower extremities. Moreover, he still had faint but generalized, well-demarcated, maculopapular rashes, but not as prominent as those found when he was admitted. Unfortunately, as the patient was admitted to a non-specialized primary hospital, a skin biopsy was not available. Furthermore, he had hematuric urine. He had intact cranial nerves and normal sensory findings. The muscles were tender and weak. Reflexes and manipulation of extremities were not done.

While the initial complete blood count (CBC) revealed slightly elevated white blood cell count (WBC; 15.5) with neutrophilic predominance (86%), the rest of the laboratory findings including dengue NS1, dengue IgG and IgM, leptospirosis IgG and IgM, and blood cultures were unremarkable. Moreover, procalcitonin was only slightly elevated [0.91 ng/ml, normal value (NV): <0.5 ng/ml], and CK MM (47 U/L, NV: <165), total (value: 54 U/L, NV: <190) and MB (value: 6.9 U/L, NV: <25 U/L) were unremarkable. Furthermore, creatinine, which was repeated several times, was within normal limits. Nevertheless, C-reactive protein (CRP, >5 mg/L) and erythrocyte sedimentation rate (ESR, 21 mm/hr, NV: <10 mm/hr) were elevated pre-and post-MPPT (CRP: 211 mg/L; ESR: 125 mm/hr). The extremely high serum ferritin (1675.56 ug/L, above the machine detection limit) clinched the diagnosis of AOSD. The patient refused to undergo nerve conduction studies (NCS)-electromyography (EMG) due to severe myalgia. He was started on daily prednisone (1 mg/kg), which led to an improvement in all symptoms. He was ambulatory and able to return to work.

## Discussion

Based on current guidelines, our patient was diagnosed with AOSD. As per Fautrel criteria, our patient fulfilled four major [quotidian fever, arthralgias, transient rash (albeit resolving when we saw the patient), PMN >80%] and two minor (maculopapular rash, leukocytosis >10000 per mm^3^) criteria; while per Yamaguchi criteria, at least five criteria were satisfied [four major (quotidian fever, arthralgias, typical rash, leukocytosis with 80% or more granulocytes) and one minor (negative tests for ANA and RF)] [1,5]. Although the glycosylated fraction of ferritin was not done, fulfillment of both criteria pegs the sensitivity and specificity of the diagnosis to 93.5% and 98.5% respectively [1,2]. Moreover, the extremely elevated serum ferritin further endorsed the diagnosis of AOSD.

Serum ferritin is a non-specific acute phase reactant notably elevated in most inflammatory conditions. This marker is produced by either activation of the histiocyte macrophage system or by the destruction of hepatocytes. In contrast to other rheumatologic conditions, serum ferritin is extremely elevated in AOSD, with levels higher than 250,000 ng/ml being reported [1]. Using a cut-off of 1000 ng/ml, it has a sensitivity of 80% and specificity of 40%, but when combined with an abnormal glycosylated fraction of ferritin, the specificity increases to around 90%, albeit, with an accompanying decrease in sensitivity (40%). As the glycosylated fraction of ferritin remains low during remission, it can be used as a marker for disease activity [1].

In the absence of necessary diagnostics in a young male patient with severe muscle pains, the urine discoloration may signify myoglobinuria. The combination of myalgia, weakness, and possible myoglobinuria may point to myositis, especially in the eyes of a neurologist [6]. Specifically, in patients with dermatomyositis, skin lesions, myalgia, myoglobinuria, and progressive weakness may also be present. Nevertheless, the normal CK-MM, creatinine and the abundance of red blood cells in the urinalysis render this less likely. Moreover, the presence of fever, transient rash, and polyarthralgia, relatively uncommon in myopathies, may hint at other possible differentials. Earlier detection of AOSD, in this case, may have saved a lot of resources, as further work-up for myositis involves costly antibody testing and unnecessary invasive muscle biopsies.

As AOSD is a multisystem condition, it is not surprising that AOSD with inflammatory myositis has already been reported. One study reported a 41-year-old female with AOSD who also developed criteria-diagnosed polymyositis, albeit without the presence of hematuria. In the same year, a similar condition was also reported in a 43-year-old male in France [7,8]. AOSD may also have renal implications. In a recent systematic review involving 36 patients with AOSD who underwent kidney biopsy, 25% had AA amyloidosis, 11.4% had collapsing glomerulopathy, and 11.4% had thrombotic microangiopathy among others; hence, hematuria among AOSD patients may not be uncommon [3]. Muscle diseases in general present with proximal weakness [4,9,10]. While our patient was bed-bound and immobile, we suspect that his limitation in movement was secondary to severe polyarthralgia and myalgia and not due to weakness. Around 64-100% and 56-84% of AOSD patients developed disabling arthralgia and generalized myalgia respectively. The swift recovery in ambulation in our patient after the administration of analgesics confirmed this hypothesis.

In terms of management, only around 12% of cases will improve after the administration of analgesic and antipyretic as monotherapy. As with other autoimmune and inflammatory conditions, steroids may be required in 57-77% of AOSD patients [1]. These have a response rate of up to 95%. Anakinra, an anti-interleukin 1 receptor antagonist, is another option for these patients [11]. For patients with severe polyarthritis, methotrexate may be given, although randomized clinical trials for this and other immunomodulatory drugs have not yet been conducted [1]. In our case, the administration of steroids led to the resolution of our patient’s myalgia and hematuria.

## Conclusions

We presented the case of a young male with AOSD mimicking a myopathy. Early detection of AOSD in such cases may save a lot of resources, as further workup for myositis involves costly antibody tests and unnecessary invasive muscle biopsies.
